# The evolutionary and phylogeographic history of woolly mammoths: a comprehensive mitogenomic analysis

**DOI:** 10.1038/srep44585

**Published:** 2017-03-22

**Authors:** Dan Chang, Michael Knapp, Jacob Enk, Sebastian Lippold, Martin Kircher, Adrian Lister, Ross D. E. MacPhee, Christopher Widga, Paul Czechowski, Robert Sommer, Emily Hodges, Nikolaus Stümpel, Ian Barnes, Love Dalén, Anatoly Derevianko, Mietje Germonpré, Alexandra Hillebrand-Voiculescu, Silviu Constantin, Tatyana Kuznetsova, Dick Mol, Thomas Rathgeber, Wilfried Rosendahl, Alexey N. Tikhonov, Eske Willerslev, Greg Hannon, Carles Lalueza-Fox, Ulrich Joger, Hendrik Poinar, Michael Hofreiter, Beth Shapiro

**Affiliations:** 1 Department of Ecology and Evolutionary Biology, University of California, Santa Cruz, Santa Cruz, CA 95064, USA; 2 Department of Anatomy, University of Otago, 270 Great King Street, Dunedin 9016, New Zealand; 3 McMaster Ancient DNA Centre, Department of Anthropology, McMaster University, 1280 Main Street West, Hamilton, Ontario L8S 4L9, Canada; 4 Department of Evolutionary Genetics, Max Planck Institute for Evolutionary Anthropology, Deutscher Platz 6, Leipzig D04103, Germany; 5 Department of Genome Sciences, University of Washington, 3720 15^th^ Ave NE, Seattle, WA 98195-5065, USA; 6 Department of Earth Sciences, The Natural History Museum, Cromwell Road, London SW7 5BD, UK; 7 Department of Mammalogy, American Museum of Natural History, 200 Central Park West, New York NY, 10024, USA; 8 Center of Excellence in Paleontology, East Tennessee State University, 1212 Sunset Dr., Gray, TN 37615, USA; 9 Antarctic Biological Research Initiative, 31 Jobson Road, SA 5110, Australia; 10 Department of Zoology, Institute of Biosciences, University of Rostock, Universitätsplatz 2, Rostock D-18055, Germany; 11 Department of Biochemistry, Vanderbilt University School of Medicine, 2215 Garland Ave, Nashville, TN 37232, USA; 12 Staatliches Naturhistorisches Museum Braunschweig, Pockelstrasse 10, Braunschweig 38106, Germany; 13 Swedish Museum of Natural History, Department of Bioinformatics and Genetics, S-104 05 Stockholm, P.O. Box 50007, Sweden; 14 Institute of Archaeology and Ethnography, Siberian Branch, Russian Academy of Sciences, 17, Novosibirsk, Akademia Lavrentieva, 630090, Russia; 15 Operational Directorate “Earth and History of Life”, Royal Belgian Institute of Natural Sciences, Vautierstraat 29, Brussels 1000, Belgium; 16 “Emil Racoviţă” Institute of Speleology, Frumoasă 31, Bucharest, 01906, Romania; 17 Department of Palaeontology, Faculty of Geology, Moscow State University, ul. Leninskiye Gory, 1, Moscow, 119991, Russia; 18 Mammuthus Club International, Gudumholm 41, Hoofddorp, HG 2133, Netherlands; 19 Staatliches Museum für Naturkunde Stuttgart Rosenstein, Gewann 1, Stuttgart 70191, Germany; 20 Department “World Cultures and Environment”, Reiss-Engelhorn-Museen, C 5, Zeughaus, Mannheim, 68159, Germany; 21 Zoological Institute Russian Academy of Sciences, Universitetskaya nab., 1 Saint-Petersburg 199034, Russia; 22 Institute of Applied Ecology of the North, North-Eastern Federal University, Lenina 1, Yakutsk, Russia; 23 Centre for GeoGenetics, Copenhagen University, Nørregade ‘10, Copenhagen, 1165, Denmark; 24 Department of Zoology, University of Cambridge, Downing St. Cambridge, CB2 3EJ, UK; 25 Sanger Institute, Wellcome Trust Genome Campus, Hinxton, CB10 1SA, UK; 26 CRUK Cambridge Institute, University of Cambridge, Robinson Way, Cambridge, CB2 0RE, UK; 27 Institute of Evolutionary Biology (CSIC-UPF), Doctor Aiguader, 88, Barcelona, 08003, Spain; 28 Department of Mathematics and Natural Sciences, Evolutionary Adaptive Genomics, Institute for Biochemistry and Biology, University of Potsdam, Karl-Liebknecht-Str. 24-25, Potsdam, 14476, Germany

## Abstract

Near the end of the Pleistocene epoch, populations of the woolly mammoth (*Mammuthus primigenius*) were distributed across parts of three continents, from western Europe and northern Asia through Beringia to the Atlantic seaboard of North America. Nonetheless, questions about the connectivity and temporal continuity of mammoth populations and species remain unanswered. We use a combination of targeted enrichment and high-throughput sequencing to assemble and interpret a data set of 143 mammoth mitochondrial genomes, sampled from fossils recovered from across their Holarctic range. Our dataset includes 54 previously unpublished mitochondrial genomes and significantly increases the coverage of the Eurasian range of the species. The resulting global phylogeny confirms that the Late Pleistocene mammoth population comprised three distinct mitochondrial lineages that began to diverge ~1.0–2.0 million years ago (Ma). We also find that mammoth mitochondrial lineages were strongly geographically partitioned throughout the Pleistocene. In combination, our genetic results and the pattern of morphological variation in time and space suggest that male-mediated gene flow, rather than large-scale dispersals, was important in the Pleistocene evolutionary history of mammoths.

Late Pleistocene mammoth remains are common across Eurasia and North America in temperate as well as high latitude areas^1^,^2^. However, until recently, little effort was applied to the recovery of ancient DNA (aDNA) from fossils collected in mid-continental areas, as preservation of genetic material in these regions is generally much poorer than in high-latitudes. Gradual improvements in methodology and instrumentation have enabled recovery of aDNA from more challenging remains, such as from mammoth bones preserved in temperate regions of Eurasia^3^,^4^,^5^ and, more recently, North America^6^. These data can be used to resolve persisting questions regarding mammoth diversity and population structure.

Our understanding of the evolutionary history of *Mammuthus* has been developed largely from morphological study of their fossils, especially the most resilient and therefore most abundant elements: molar teeth^1^,^7^,^8^,^9^,^10^. According to the fossil evidence, mammoths evolved in Africa during the late Miocene and later dispersed into Asia and Europe, and eventually North America via Beringia, during the Middle Pliocene to Early Pleistocene^1^,^10^,^11^. The evolution of *Mammuthus* during the Pleistocene is usually presented as a succession of chronologically overlapping species, including (from earliest to latest) *M. meridionalis* (southern mammoths), *M. trogontherii* (steppe mammoths), and *M. columbi* (Columbian mammoths) and *M. primigenius* (woolly mammoths)^1^,^7^,^8^,^10^.

According to the current model based on morphology, a population of southern mammoths gave rise to the steppe mammoth around ~1.7 million years ago (Ma) in Asia. Later, perhaps as early as 0.7 Ma, a second transition occurred in Asia as a steppe mammoth population gave rise to the woolly mammoth^1^,^12^. Subsequently, these species dispersed out of Asia into Europe and North America. The European fossil record suggests several distinct waves of dispersal into Europe, after which the migrants may have coexisted and even hybridized with endemic European mammoth populations^2^,^8^. Until recently, it was generally held that southern mammoths dispersed into North America around 1.5 Ma^13^, where they evolved into Columbian mammoths (*M. columbi*), Jefferson’s mammoth (*M. jeffersonii*) and the Channel Islands pygmy mammoth (*M. exilis*)^6^,^7^. However, Lister and Sher^2^ recently suggested that southern mammoths never migrated to North America, and that all early North American mammoth fossils (1.5–1.3 Ma) that are classifiable based on morphology descend from dispersal(s) of steppe mammoths. A key implication of this interpretation is that the steppe mammoth population in northeastern Siberia was ancestral to both Columbian and woolly mammoths although at different points in time. Results of a recent study^6^ analysing complete mitogenomes from North American mammoth populations were consistent with this hypothesis. Furthermore, this study found evidence of hybridisation and potential male mediated gene flow between North American woolly mammoths, Columbian mammoths, Jefferson’s mammoth and pygmy mammoth^6^.

Eurasian mammoths have received far less attention thus far. Previous population-level genetic analyses beyond the permafrost regions of Western Beringia were restricted to a small fragment of the mitochondrial genome. These studies partitioned woolly mammoth mitochondrial diversity into either three major clades^3^,^5^ or five haplogroups^4^ but were unable to resolve either the order in which these clades emerged or the timing of their origin. As a result the evolution of mammoths across their vast Eurasian range remains unclear.

Here, we combine hybridization-based targeted capture^14^,^15^,^16^, multiplex PCR^17^,^18^ and high-throughput sequencing to generate 54 complete mammoth mitochondrial genomes, including 22 from temperate localities across Eurasia. We incorporate these into a globally extensive data set totalling 143 mammoth mitochondrial genomes sampled from across the northern hemisphere. We use a combined approach that incorporates both the deep paleontological record and more recent radiocarbon dates to calibrate the evolutionary rate, and infer the most comprehensive mammoth mitochondrial phylogeny to date.

## Results

**Figure 1:**
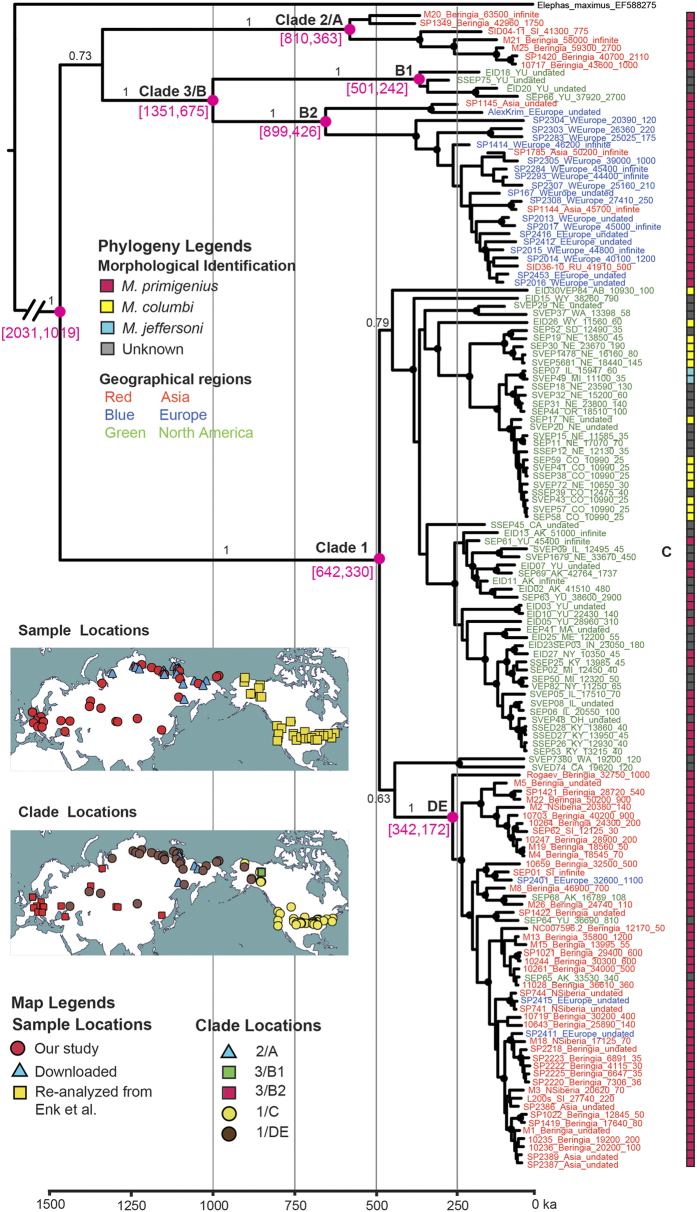
Maximum Clade Credibility (MCC) phylogeny resulting from a BEAST analysis of the *relaxed* data set. The molecular clock was informed using the root-and-tip-dating method. Morphology-based taxonomic identifications are provided as rectangular bars to the right of sample names. Nodes leading to major clades are labeled and highlighted as magenta dots, with the inferred tMRCA (95% highest posterior density of node ages, magenta numbers below the branch leading to the clade). Posterior probability for each clade is provided either as black numbers along branch leading to major clades, or indicated as >95% by the presence of a black dot. The timescale is offset by 4651 years, which is the age of the youngest sample in the data set. (Inset) Maps describing the geographic location of (a) samples used in this study and (b) the clade and haplogroup information of those samples. Map from http://www.d-maps.com/carte.php?num_car=3212&lang=en.

Using hybridization capture combined with high-throughput sequencing, we generated at least one-fold coverage of the complete mitochondrial genomes from 54 of 63 mammoth bones that we collected from sites across Eurasia and North America (Fig. 1 inset; Supplementary Materials). In addition, we reassembled raw data from 79 North American mammoth mitochondrial genomes^6^ following the same bioinformatics pipeline as used to assemble our new genomes. For each sample, de-multiplexing, mapping and duplicate removal resulted in between 367 and 211,184 unique mitochondrial reads, which is equivalent to 1-fold to 819-fold coverage of the mitochondrial genome (Table S1). To evaluate potential error associated with missing and low-coverage data and to include the largest number of individuals possible, we adopted two strategies for consensus calling and filtering (see Methods) that resulted in two data sets: a *relaxed* alignment of 117 mammoth mitochondrial genomes and a *strict* alignment of 70 mitochondrial genomes. To both of these, we added 17 previously published mitochondrial genomes^18^,^19^,^20^,^21^ and nine sequences obtained using a multiplex PCR and pyrosequencing approach (Supplementary Materials), for a total of 143 mitochondrial genome sequences in the *relaxed* alignment and 96 in the *strict* alignment.

Figure 1 shows the maximum clade credibility (MCC) mammoth mitochondrial genealogy resulting from a Bayesian phylogenetic analysis of the *relaxed* data set, using a strict molecular clock and the root-and-tip calibration approach; the results from the strict data set are provided in Figure S2. Consistent with previous studies, mammoth mitochondrial variation is represented by three major lineages^5^,^6^. Unlike in most previous studies, these three lineages are strongly statistically supported, regardless of data set or rate calibration approach.

The three main mammoth mitochondrial clades shared a most recent common ancestor (MRCA) ~2.0–1.0 Ma (Fig. 1). Clade 3 (haplogroup B) shows the deepest divergence of the three lineages, with a MRCA ~1.4–0.7 Ma. This lineage has been referred to in the past as the European clade^5^. However, we find strong support for a genetically distinct North American component to this lineage in Alaska/Yukon, which we therefore subdivide into haplogroups B1 (exclusively eastern Beringian) and B2 (European and Siberian). Clade 2 (haplogroup A) probably originated in Asia and shares a MRCA ~810–360 ka. Clade 2 appears to have been geographically restricted to western Beringia and northern Siberia until its extinction. Clade 1 mammoths share a MRCA ~640–330 ka, and include previously defined haplogroups C, D and E^4^.

## Discussion

### Mammoth mitochondrial diversification and correlation with the fossil record

Previous population-level analyses of mammoth mitochondrial diversity have provided conflicting estimates of the timing of mammoth mitochondrial diversification, with estimates of the MRCA of all mammoth mitochondria ranging from within the last 300,000 years^5^ to more than 2 million years ago^20^. Often, divergence estimates result from analyses in which the evolutionary rate is calibrated using only the ages of each sampled mammoth^3^,^5^. When multiple calibrations are used, the resulting divergence estimates are much older^4^,^20^. We adopt the calibration approach of Enk *et al*.^6^, in which we calibrate the evolutionary rate using both the age of each sampled mammoth and a 6.7 Ma divergence between Asian elephants and mammoths^22^. As the Asian elephant/mammoth divergence is much more recent than the elephant/mastodon divergence used previously^4^,^20^, this calibration reduces the impact of long-term fixation and saturation of mutations.

**Figure 2:**
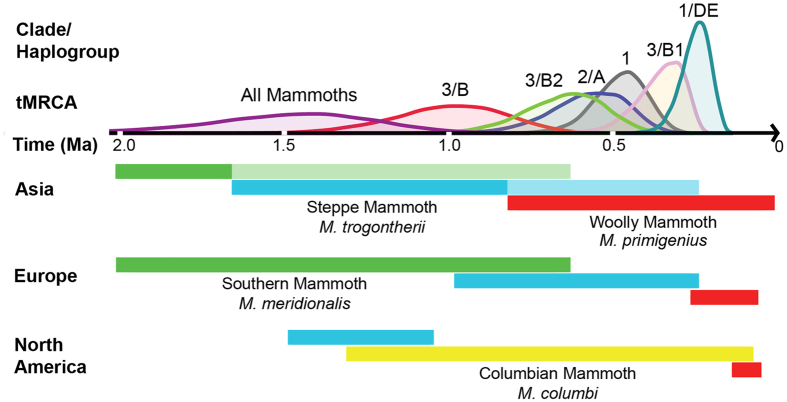
Comparisons of tMRCAs of major clades as inferred from the mitogenomic data presented in Fig. 1 and the paleontological record of mammoth evolution. Above the time axis are the posterior distributions of tMRCAs of major clades estimated with the root-and-tip-dating method. Color bars below the time axis represent paleontological records of the named mammoth species in Asia, Europe and North America. In Asia, shaded bars reflect that last occurrence dates for Southern and Steppe mammoths are uncertain.

After establishing a time-calibrated global phylogeny of mammoth mitochondrial evolution, we compared it to some of the patterns observed in the fossil record. Figure 2 provides an overview of both the mitochondrial data presented in Fig. 1 and the morphological data from the fossil record.

First, we consider the transition from southern mammoth (Fig. 2, green bars) to steppe mammoth (Fig. 2, blue bars). The paleontological record indicates that southern mammoths were widespread across the mid-latitudes of Eurasia at the upper end of the time range of the inferred MRCA of all sampled mammoths^1^. In eastern Asia, the paleontological transition from southern mammoth to steppe mammoth morphology occurs *ca.*1.7–1.6 Ma^1^,^12^. In Europe, the replacement of southern by steppe mammoth occurs *ca.* 1.0–0.6 Ma^8^, which coincides with our estimated MRCA of clade 3. Therefore, we suggest that the dispersal of clade 3 mammoths into Europe may be associated with the paleontological transition between southern and steppe mammoth.

Second, our evaluation of mammoth migration into North America based on our global mammoth phylogeography confirms results from Enk *et al*.’s study of North American mammoths^6^. Recent re-evaluation of the North American paleontological record^2^ rejected previous hypotheses that the southern mammoth was common in North America, and instead assigned all identifiable specimens to either Columbian mammoth, woolly mammoth or steppe mammoth. Based on our data, the dispersal of clade 3 mammoths into North America must have occurred at the latest by ~500–240 ka (the MRCA of haplogroup B1) but possibly as early as ~1.3 Ma (the upper end of the MRCA of haplogroups B1 and B2). However, even the oldest dates in this range are marginal to the earliest mammoths in North America, which are dated to 1.3–1.5 million years. Instead, the dispersal of steppe mammoths into North America may be represented in our data set by the primarily North American clade 1, which shares a MRCA 330–640 ka, but which had already diverged from the other two major mitochondrial clades by ~1.5 million years ago. This earlier divergence provides an excellent fit to the first appearance of mammoths in North America^2^. Since all *M. columbi* individuals sequenced thus far belong to clade 1, it is likely that clade 1 mammoths represent the ancestors of Columbian mammoths in North America.

Third, we consider the transition from the steppe mammoth to woolly mammoth (Fig. 2, red bars). Morphological features associated with woolly mammoths appeared in Asia ~800–600 ka but much later in Europe, ~0.2 Ma^1^; and in North America ~125 ka^2^,^6^. We do not observe a corresponding mitochondrial replacement either in Europe or in North America. Instead, the MRCA of clade 1, which originated in North America (Fig. 2 4); significantly predates this morphological transition in North America (Fig. 2).

It is important to note that the timing of population divergence does not necessarily coincide with the timing of divergence among mitochondrial lineages. Our interpretations are therefore speculative to some extent. However, we feel that the notable overlap between morphological divergence and the timing of divergence estimated from these mitochondrial data justifies the discussion presented here. Future research using nuclear genomic data will no doubt clarify these relationships further.

### Reconciling morphological similarity and diversity with mitochondrial structure

While the genes encoded by mitochondrial DNA cannot be responsible for the morphological transitions observed in the fossil record, the geographic and temporal partitioning of mitochondrial diversity can provide new insights into mammoth population dynamics. Figure 1 shows that mammoth mitochondrial lineages were strongly geographically partitioned throughout the Pleistocene, with infrequent mitochondrial gene flow across the Bering Land Bridge and almost no mitochondrial gene flow into Europe after the establishment of clade 3. Previous work^5^ indicates that the transition to mitochondrial clade 1 in Europe took place very late in the Pleistocene, around 30,000 years ago.

Late Pleistocene Eurasian and some North American mammoths exhibit morphological characteristics that clearly identify them as woolly mammoths. Despite these similarities, they represent three distinct mitochondrial lineages and more than one million years of mitochondrial evolution (Fig. 1). This suggests either that only limited gene flow was necessary to homogenize the populations across large distances, or that more extensive gene flow was occurring between geographic regions, but that dispersal was restricted mainly, although not entirely, to males, transmitting nuclear DNA and hence most phenotypic characters. Late Pleistocene mammoths in Europe show a wider range of variation than those in Beringia, encompassing both steppe and woolly mammoth morphotypes^2^,^8^. This is consistent with predominately male dispersal of woolly mammoths from Beringia to Europe after 200 ka (see above) where they encountered and presumably hybridised with clade 3 steppe mammoths. In North America, the presence of both Columbian and woolly mammoths in mitochondrial clade 1^6^, which originated and diversified within North America, similarly supports the hypothesis of male-mediated gene flow. In this case, it is possible that dispersing male woolly mammoths hybridized with female Columbian mammoths, leading to the mitochondrial structure observed in Fig. 1 and to morphological diversity including North American nominal mammoth species^2^. It is therefore likely that a similar mechanism explains the North American Late Pleistocene mammoth complex and the morphological transition from steppe mammoth to woolly mammoth in Europe^2^,^6^.

Differential dispersal between sexes has been widely documented in African elephants^23^,^24^. Female philopatry leads to strong mitochondrial population structure among these elephants, while male-mediated dispersal is known to homogenize populations with respect to both morphology and nuclear genetic differentiation^24^,^25^. In particular, African elephants are classified into forest and savanna species (*Loxodonta cyclotis* and *L. africana*). Forest and savanna elephants live in different habitats, have different morphologies, and are highly divergent at the nuclear genetic level^25^,^26^,^27^,^28^,^29^. The mitochondrial diversity of all African elephants is partitioned into two clades, F and S, that diverged ~5.5 Ma. Individuals of the forest species all have the F mitochondrial lineage, whereas those of the savanna species have either S or F mitochondrial lineages^26^,^30^. It has been hypothesized that the morphological and nuclear similarities among savanna elephants are maintained by male-mediated gene flow, while female philopatry allows the two deeply diverged mitochondrial lineages to persist^28^.

Brandt *et al*.^27^ investigated whether this mechanism might also apply to mammoths. They argued that, for a population without sex biases in dispersal, the effective population size estimated from the mitochondrial DNA should be ~25% of the size estimated from the nuclear genome. But for a species that exhibits strong female philopatry, the ratio of coalescent time estimated from mitochondrial DNA to that of the nuclear loci should be higher than 0.25. They estimated the coalescent date from two mitochondrial genomes representing Clade 1 and 2^18^,^20^, and compared it with the coalescent time estimated from more than 300 nuclear loci^29^. The ratio of mitogenome to nuclear dates for mammoths is significantly higher than 0.25 (this is also observed by ref. 31), similar to the pattern observed in African and Asian elephants, supporting the hypothesis of female philopatry and male-mediated gene flow in mammoths.

The regular dispersal of a subset of mammoth populations is also supported by North American isotopic data on tooth enamel. Although groups of mammoths typically share isotopic values suggesting shared diet and mobility histories, a small proportion of individuals, possibly males, show distinct values suggesting movements from 150–600 km^32^,^33^. This would be roughly consistent with median (~144 km) and maximum (>800 km) dispersal distances calculated on the basis of the body size of an average mammoth (~5000 kg)^34^.

It is worth noting that male-mediated gene flow from woolly mammoths into steppe and Columbian mammoths represents a slightly different pattern than that observed from savannah elephants into forest elephants. In the latter, large savannah males outcompete smaller forest males. In contrast, woolly mammoth were smaller than both steppe mammoths and Columbian mammoths. However, if the steppe and Columbian mammoth phenotypes resulting from woolly mammoth admixture conferred a selective advantage, occasional interbreeding between the species may have been sufficient to spread the woolly mammoth phenotype into steppe and Columbian mammoth populations. Evidence supporting this comes from human genomic data, which indicate that presumably advantageous Neanderthal gene variants spread and persisted in the modern human gene pool despite infrequent admixture^35^,^36^.

Although our mitochondrial data indicate that female dispersal occurred infrequently in mammoth evolutionary history, we nonetheless find evidence of several episodes of female dispersal. For example, the first appearance of clades B2 in Europe and B1 in North America probably reflect female dispersal from Asia, although only the former can tentatively be associated with a morphological transition. The initial colonization of North America by mammoths must have occurred via both female and male dispersal, and is probably represented in our data set by clade 1. Finally, female dispersal from North America into Eurasia is indicated by the first appearance of clade 1D/E in Eurasia, perhaps followed by continued gene flow across the Bering Land Bridge.

Our analyses of 143 complete mitochondrial genomes of mammoths representing their global range of distribution provide the most complete picture of mammoth evolution to date. The complete mitochondrial genomes generated here demonstrate the power of using targeted enrichment and high-throughput sequencing approaches to obtain high quality ancient genomes for phylogeographic and population studies. The evolutionary pattern revealed by our genetic data, combined with paleontological records, suggests a mechanism of female philopatry combined with male-mediated gene flow that drives the geographical pattern of morphological variation, and provides a working hypothesis that can be tested in the future using mammoth nuclear genomes.

## Methods

### Sample collection

Over several decades, we and collaborators have collected large numbers of mammoth remains from sites across Eurasia and North America. From these, we selected 63 to be included in this study, focusing on geographic regions that have been underrepresented in previous work (Fig. 1). When possible, we identified each of these to species level based on morphological analysis. We then generated complete mitochondrial genome sequences using one of or both approaches described below (Supplementary Materials). In addition, we obtained Accelerator Mass Spectrometry (AMS) radiocarbon dates for 30 samples for which no stratigraphic information was available. The ages of each specimen included in our global data set (either from radiocarbon or stratigraphic context) are provided in Supplementary Materials.

### DNA extraction

We extracted genomic DNA from 150–250 mg of bone and tusk samples of mammoths using the approaches described in Rohland and Hofreiter^37^ and Rohland *et al*.^38^. To avoid environmental contamination, we performed extractions and subsequent experiments in a designated ancient DNA laboratory, following strict ancient DNA protocols. To avoid loss of information from molecules with deaminated bases, we did not treat DNA extracts with UDG. Instead, we use two different base-calling schemes (see below) to compensate for the presence of deaminated bases.

### Data Generation Method 1: Multiplex PCR and pyrosequencing

For ten samples (Supplementary Materials), we generated complete mitochondrial genomes following the 2-step multiplex PCR^17^, PTS tagging^39^ and Roche 454 pyrosequencing approach described in Rohland *et al*.^22^. In brief, we divided 78 primer pairs that span the mammoth mitochondrial genome into two, non-overlapping pools of 39 primer pairs. We performed multiplex PCR with each primer pool twice to generate independent replicates for each sample. Products of the four multiplex PCR amplifications then served as respective templates for singleplex amplifications with each of the 78 primer pairs. We purified, quantified and pooled the amplified mitochondrial genome fragments in equal ratios, and built tagged/barcoded 454 sequencing libraries following Meyer *et al*.^39^. After sequencing, we de-multiplexed sequences of each replicate of every sample and removed sequencing adapters. We mapped reads against a previously published mammoth mitochondrial genome (GenBank accession number EU153444)^20^ using MIA^40^. Then, we generated consensus sequences of the mitochondrial genome for each sample by retaining the majority base at each site from all reads of both replicates. We filled in gaps in the consensus sequences with additional PCR amplification and Sanger sequencing. As a positive control, we re-sequenced sample 10643 (Supplementary Materials) using the hybridization capture and Illumina high-throughput sequencing approach described below. Both approaches yielded identical consensus mitochondrial genome sequences for this sample.

### Data Generation Method 2: Enrichment via hybridization capture

For the remaining 53 samples, we attempted to generate complete mitochondrial genomes using a hybridization capture approach. Following DNA extraction, we prepared barcoded Illumina sequencing libraries following a protocol modified from Meyer and Kircher^41^ for highly degraded DNA templates^42^. We enriched each barcoded library for mammoth mitochondrial sequences using an early version of the Agilent on-array hybridization capture approach^16^. We designed 60 bp baits with 30 bp tiling from a published mammoth mitochondrial genome (GenBank accession number EU153444). Libraries of 54 Eurasian samples (53 unprocessed and one replicate as described above; marked with asterisks in Table S1) were pooled and captured on Agilent capture arrays. Finally, we sequenced the enriched libraries on the GAIIx (2 × 76 bp) at the Max Planck Institute for Evolutionary Anthropology.

### Read processing

Next, for data generated using both of the methods described above, we trimmed adaptors, filtered and retained reads with no more than five bases whose quality scores are below 10, and merged overlapping reads. We then mapped paired-end reads and merged reads to a reference mammoth mitochondrial genome using BWA^43^, with the following parameters: 0.1% probability of missing alignments with an error rate of no more than 2%, up to four gaps open, reduced gap penalties, gaps of up to two bases allowed at the end of each read, and no seed (-n 0.001 -o4 -O8 -E2 -i2 -I 65536).

Pooling libraries and performing capturing in a single reaction imposes a risk of barcode bleeding among samples. To eliminate the impact of barcode bleeding, we developed a conservative filtering approach to de-multiplex our samples in which we retained reads that met the following criteria: (a) minimum read lengths of 30 base pairs; (b) sequences with the same start and end coordinates observed at least three times; (c) a sequence cluster retained for the most frequent barcode across an experimental batch, or discarded if equally frequent for multiple barcodes.

Next, we removed PCR duplicates in each BAM file with the script *FilterUniqueBAM.py* (available from https://github.com/Paleogenomics/DNA-Post-Processing.git). This script retains a single consensus sequence for reads with the same start and end coordinates. For samples for which multiple libraries and/or capture experiments had been performed, we combined mapped reads of the same sample with SAMtools *merge*^44^ and removed duplicates with *FilterUniqueBAM.py* as described above.

### Generating consensus sequences with two filtering criteria

To control for damage-derived substitutions and sequencing error, we applied two sets of criteria to create consensus sequences and alignments from each filtered and de-duplicated library. Our *relaxed* alignment is generated using a less strict set of criteria for base calling and inclusion in the final sequence alignment: bases are called at sites with at least three non-duplicate reads and where the majority base is present with a frequency higher than 33% (at least two of every three bases in agreement). Other bases are called as an *N.* In addition, only sequences for which no more than 33% of bases can be called as *N* are included in the alignment. The *strict* alignment was generated by requiring at least 10-fold coverage at each site and that the majority base is present in more than 90% of reads. To be included in the sequence alignment, no more than 20% of sites can be missing in the resulting consensus sequence. We used SAMtools *mpileup* and BCFtools to obtain coverage and variant information, and developed a script *VCF2consensus.py* (also available from https://github.com/Paleogenomics/DNA-Post-Processing.git) to generate consensus sequences with the two filtering criteria described above. We also included nine sequences that were obtained through multiplex PCR and pyrosequencing as described above into the two alignments. We did not include the multiplex PCR results of sample 10643 because the mitochondrial genome of this sample was also generated with hybridization capture and has been included in each alignment.

### Data from other studies

We also included in our study 79 North American mammoth mitochondrial genomes that were generated recently^6^ (Supplementary Materials). To provide consistency between these and our data, we obtained raw sequencing libraries from each of these 79 samples (GenBank accession numbers KX027489-KX027568), and used the read processing, de-multiplexing and filtering approaches described above to generate consensus sequences.

Finally, we also downloaded 17 previously published complete mitochondrial genome sequences (GenBank accession numbers EU153445-EU153458, NC007596 and DQ316067; Supplementary Materials)^18^,^19^,^20^,^21^. These mitochondrial genomes were included as published in both the *relaxed* and *strict* data sets.

### Alignment, sequence partition and model selection

We aligned each data set using Seaview v4.0^45^ with the algorithm *muscle –maxiters2 –diags*, and adjusted alignments manually in Se-Al v2.0^46^. For both data sets, we created four sequence partitions: protein-coding genes, the control region, transfer RNA (tRNA) and ribosomal RNA (rRNA). We reverse-complemented genes in the reverse strand, and concatenated genes and regions of the same partition category with Biopython^47^. We performed model selection for partitions of the control region, tRNA and rRNA separately in jModelTest 2.1.3^48^, selecting the best model for each partition from full models with gamma distribution (+G), invariable sites (+I) and equal/unequal base frequencies. The best models suggested by the Bayesian Information Criterion are HKY (Hasegawa, Kishino and Yano) +I+G model^49^ for the control region, and the HKY +I model for rRNAs and tRNAs respectively. We chose the SRD06 model^50^ for the partition of protein-coding genes because this model effectively captures the substitution patterns of different codon positions.

### Inferring phylogenetic relationships

To assess whether the inferred mitochondrial phylogeny and divergence estimates are robust to differences in coverage, variant calling and missing sites, we performed Bayesian phylogenetic analyses on the *strict* and *relaxed* alignments using BEAST v1.8.0^51^. To calibrate the evolutionary rate, we first used the age of each sequence as prior information in a tip-dating approach^52^. Age information was incorporated either as mean calibrated radiocarbon dates (for specimens with finite radiocarbon dates <41,000 years old) or sampled from a prior distribution with a wide range based on both stratigraphic information and prior genetic studies (a lognormal distribution with a mean of 50,000 years and a range of 10,000 to 270,000 years^5^). We calibrated ages of finite samples in calendar years before present, using OxCal v4.2^53^ and the intCal13 Northern Hemisphere atmospheric radiocarbon calibration curve^54^. To compensate for violating the assumption of sampling from a single population, we used a flexible coalescent prior (the Bayesian Skyline Model^55^) and an uncorrelated lognormal relaxed clock^56^. Model comparison^57^ revealed, however, that the constant population size model fit the data slightly better than the flexible model, and that the strict clock model was a better fit for the tRNA partition. Our final analyses incorporated the models supported by model tests.

For each BEAST analysis, we ran two independent MCMC chains of 100 million generations each, sampling trees and model parameters every 10,000 generations. We inspected the combined results in Tracer v1.6^58^, and determined convergence of each parameter, all of which had their ESS values larger than 200. We identified the Maximum Clade Credibility (MCC) tree in TreeAnnotator v1.8.0, and visualized and graphically edited the MCC tree using FigTree v1.4.0^59^. The resulting phylogenies for the *strict* and *relaxed* data sets were similar both in topology and timescale (Figs S1 and S2).

It has been observed that estimated evolutionary rates can differ significantly depending on calibration approach, with faster rates estimated from relatively shallow timescales and slower rates estimated from deeper timescales. Based on the fossil record, tip-only calibrations are likely to be inappropriate for our data set^60^,^61^. We therefore performed additional analyses on the *relaxed* data set in which we incorporated both the radiocarbon-dated tips and a calibration point that lies much deeper within the tree to infer the evolutionary rate (the root-and-tip-dating method)^6^,^62^. We did not perform this dating on the *strict* data set, which did not include the oldest (and least well-preserved) mammoth samples. We aligned the *relaxed* data set to the complete mitochondrial genome of an Asian elephant (GenBank accession number EF588275), and assumed a normally distributed divergence time between the Asian elephant and mammoths of 6.7 million years with a standard deviation of 0.5 million years^22^. Model testing and BEAST analyses were performed as described above. As expected, rates estimated from the *relaxed* data set were approximately two times faster when only the tip calibration was used compared to the root-and-tip-dating approach (Fig. S3).

To determine the clade and haplogroup designation of our new mammoth sequences, we downloaded partial mitochondrial sequences published previously (GenBank Accession numbers: FJ015093–FJ015152; KC427894-KC427981)^3^,^4^,^5^. We aligned downloaded sequences with our complete mitochondrial genomes in Seaview^45^ as described above, and built a neighbor-joining tree with 100 bootstrap replicates. The classification of clades was based on similarity and phylogenetic grouping of our sequences with published partial mitochondrial sequences of known clades/haplogroups.

## Additional Information

**Accession Codes:** Mitochondrial genome sequences generated in this study have been deposited in GenBank (accession numbers KX176750 - KX176803).

**How to cite this article:** Chang, D. *et al*. The evolutionary and phylogeographic history of woolly mammoths: a comprehensive mitogenomic analysis. *Sci. Rep.* **7**, 44585; doi: 10.1038/srep44585 (2017).

**Publisher's note:** Springer Nature remains neutral with regard to jurisdictional claims in published maps and institutional affiliations.

## Supplementary Material

Supplementary Information
