# Supplementary material for: The evolutionary and phylogeographic history of woolly mammoths: a comprehensive mitogenomic analysis
Source: Sci Rep. 2017 Mar 22;7:44585. doi: 10.1038/srep44585 (PMC5361112; doi:10.1038/srep44585)
Supplement: Supplementary Information [file srep44585-s1.pdf]

Supplementary Material
for
The evolutionary and phylogeographic history of woolly mammoths: a comprehensive mitogenomic analysis

Dan Chang*, Michael Knapp*, Jacob Enk*, Sebastian Lippold, Martin Kircher, Adrian Lister, Ross D. E. MacPhee, Christopher Widga, Paul Czechowski, Robert Sommer, Emily Hodges, Nikolaus Stümpel, Ian Barnes, Love Dalén, Anatoly Derevianko, Mietje Germonpré, Alexandra Hillebrand-Voiculescu, Silviu Constantin, Tatyana Kuznetsova, Dick Mol, Thomas Rathgeber, Wilfried Rosendahl, Alexey N. Tikhonov, Eske Willerslev, Greg Hannon, Carles Lalueza-Fox, Ulrich Joger, Hendrik Poinar, Michael Hofreiter, Beth Shapiro.

Table S1-S4 provide details of all samples used in the different extraction/library preparation schemes. Table S5 provides details of the *strict* and *relaxed* analyses.

Table S1: Samples prepared using method 1, multiplex PCR

ID	Region	Location	Museum Number	Latitude	Longitude	Radiocarbon Number	14C date	Sigma	intCal13 Median	Radiocarbon Date Reference	GenBank Acc.
10235	Russia	Bykovsky Peninsula	MKh-O621	73.00	128.50	GIN 10235	19200	200	23138	Barnes_et al. 2007	KX176795
10236	Russia	Bykovsky Peninsula	MKh-O422	71.79	129.40	GIN 10236	20200	100	24269	Barnes_et al. 2007	KX176796
10244	Russia	Bykovsky Peninsula	Mkh-O433	71.79	129.40	GIN 10244	30300	600	34376	Barnes_et al. 2007	KX176797
10247	Russia	Bykovsky Peninsula	MKh-O429	71.79	129.40	GIN 10247	28900	200	33101	Barnes_et al. 2007	KX176798
10261	Russia	Bykovsky Peninsula	MKh-O533	71.79	129.40	GIN 10261	34000	500	38456	Barnes_et al. 2007	KX176799
10264	Russia	Bykovsky Peninsula	MKh-O381	71.79	129.40	GIN 10264	24300	200	28332	Barnes_et al. 2007	KX176800
10643	Russia	Wrangel Island	ILC.08(10643)	71.00	179.00	OxA 11841	25890	140	30131	Barnes_et al. 2007	KX176750
10659	Russia	Bolshoy Lyakhovsk(i)y Isl.	BL-O865	73.33	141.40	GIN 10659	32500	500	36571	Barnes_et al. 2007	KX176801
10703	Russia	Bolshoy Lyakhovsk(i)y Isl.	BL-O585	73.34	141.31	GIN 10703	40200	900	43927	Barnes_et al. 2007	KX176802
11028	Russia	Bolshoy Lyakhovsk(i)y Isl.	ILC.04(11028)	74.00	138.00	OxA 11748	36610	360	41233	Barnes_et al. 2007	KX176803

Table S2: Samples prepared using method 2, mitochondrial capture

ID	Region	Location	Museum Number	Latitude	Longitude	Radiocarbon Number	14C date	Sigma	intCal13 Median	Radiocarbon Date Reference	GenBank Acc.
10643	Russia	Wrangel Island	ILC.08(10643)	71.00	179.00	OxA 11841	25890	140	30131	Barnes_et al. 2007	KX176750
10717	Russia	Bolshoy Lyakhovsk(i)y Isl.	BL-O308	73.34	141.31	GIN 10717	43600	1000	47022	Barnes_et al. 2007	KX176751
10719	Russia	Yakutia, Lena Delta Region	Nag-99-O203	72.90	123.35	GIN 10719	30200	400	34261	Barnes_et al. 2007	KX176752
AlexKrim	Ukraine	Emine Bair Khosar, Krim Peninsula		44.80	34.29						KX176785
SP1021	Russia	Bykovsky Peninsula	MKh-O438	71.79	129.40	GIN 10245	29400	600	33499	Barnes et al. 2007	KX176753
SP1022	Russia	Bykovsky Peninsula	MKh-O435	71.79	129.40	CAMS 158460/ GIN 10242	12845	50	15312	This study	KX176754
SP1144	Russia	Strashnaya cave, Altai		51.08	83.03	CAMS 158463	45700	infinite		This study	KX176767
SP1145	Russia	Ust Kanskaya-Cave 4km southeast of village Ust-Kan, on the right bank of the Tcharysh river (Tschrysch river). On the Beliy Kamen (White Stone Mountain), Altai.		50.92	84.78						KX176768
SP1349	Russia	Bolshaya Kolopatkaya river, Kolyma Lowland, Yakutia Sakha, Siberia, Russia, Very close to Cherskii, at a small river called Big grouse river.		68.733	161.383	KIA 27805	42960	1750	46623	Roempler et al. 2006	KX176755
SP1414	Germany	Siegsdorf, Germany		47.82	12.64	OxA-30164/ KIA 14407	>46200/ 45180	+1130 /-990		Rosendahl et al. 2005 (KIA date)	KX176769

SP1419	Russia	Mammontoviy Klyk, Olenek-Anabar Reg., Siberia	MaK-O101	73.61	117.13	CAMS 158459	17640	80	21334	This study	KX176756
SP1420	Russia	Oyagossky Yar, Siberia	NS-OgK-O271	72.68	143.52	KIA-27803	40700	+2110 /-1670	44773	Rompler et al. 2006	KX176757
SP1421	Russia	Bykovsky Peninsula, Siberia	MKh-O468	71.81	129.35	KIA-27804	28720	+540/-500	32749	Rompler et al. 2006	KX176758
SP1422	Russia	Bolshoy Lyakhovskiy Isl., Siberia	BL-O600-Z	73.32	141.37						KX176759
SP1584	Russia	Wrangel Island	LabNo 811	71.28	-178.89	OxA-30170	28690	290	32765	This study	
SP167	Germany	Herne West, Germany		51.54	7.20						KX176770
SP1781	China	Hongqi site near Harbin, river bed, (2nd site), Heilongjiang Province, China		45.74	126.66						
SP1785	China	Yong an chun site near Harbin, river bed, (1st site), Heilongjiang Province, China		45.74	126.66	OxA-30165	>50200			This study	KX176771
SP2013	Europe	North Sea	trenched 20/08/2007	56.51	3.52						KX176772
SP2014	Europe	North Sea	trenched 28/07/2007	56.51	3.52	OxA-30167	40100	1200	43960	This study	KX176773
SP2015	Europe	North Sea	trenched 28/08/2007	56.51	3.52	OxA-30168	>44800			This study	KX176774
SP2016	Europe	North Sea	trenched 28/07/2007	56.51	3.52	CAMS 158600				This study	KX176775
SP2017	Europe	North Sea	trenched 27/08/2007	56.51	3.52	OxA-30169	>45000			This study	KX176776
SP2218	Russia	Wrangel Island	DM 13	71.28	-178.89						KX176760
SP2220	Russia	Lemming River, in the north of the Wrangel Island		71.30	-179.50	OxA-30171	7306	36	8106	This study	KX176761
SP2222	Russia	Wrangel Island	DM 18	71.28	-178.89	CAMS 158465	4115	30	4641	This study	KX176762
SP2223	Russia	Femur Station 10 (left), Wrangel Island	DM 8	71.28	-178.89	OxA-30172/30173	6891/ 6876	35/34	7720/ 7705	This study	KX176763
SP2225	Russia	Niesv. River IV, Wrangel Island	DM 32	71.28	-178.89	OxA-30174	6647	35	7529	This study	KX176764
SP2283	Germany	Saulgau, Germany		48.01	9.50	HV 25826	25025	175	29068	This study	KX176777

SP2284	Germany	Schwenningen, Germany		48.06	8.50	OxA-30179	>45400			This study	KX176778
SP2293	Belgium	Belgium	L212	50.50	4.47	CAMS 158464				This study	KX176779
SP2299	Spain	Spain	AAL/68-59.973	40.46	-3.75						
SP2300	Spain	Spain	AAL/68-60568-1	40.46	-3.75						
SP2303	Germany	Dresden-Prohlis, Germany	SaQ204	51.00	13.79	OxA-30175	26360	220	30646	This study	KX176780
SP2304	Germany	Dresden, Germany	SaQ431	51.05	13.74	OxA-30176	20390	120	24511	This study	KX176781
SP2305	Germany	Pima, Germany	SaQ18	50.96	13.97	OxA-30137	39000	1000	43057	This study	KX176782
SP2307	Germany	Gollwitz, Poel Island, Germany		54.02	11.48	KIA 35747/ OxA-30177	23900/ 25160	130/ 210	27943/2 9208	Sommer & Benecke 2009	KX176783
SP2308	Germany	Schieritz/ Lommatzsch, Germany	SaQ746	51.20	13.39	OxA-30178	27410	250	31278	This study	KX176784
SP2386	Russia	Malta, Russia	n.a	52.84	103.52						KX176786
SP2387	Russia	Malta, Russia	n.a	52.84	103.52						KX176787
SP2389	Russia	Malta, Russia	n.a	52.84	103.52						KX176788
SP2401	Russia	Kostenki (Kostyonki?)	32572 (3)	51.38	39.05						KX176789
SP2402	Russia	Kostenki	32572 (4)	51.38	39.05						
SP2411	Russia	Secrets Cave, M. Ural	31615 (2)	59.42	57.75						KX176790
SP2412	Russia	Secrets Cave, M. Ural	31615 (3)	59.42	57.75						KX176791
SP2415	Russia	Secrets Cave, M. Ural	31615 (9)	59.42	57.75						KX176792
SP2416	Russia	Medvezhiya Cave, N. Ural	34760	62.00	58.73						KX176793
SP2453	Ukraine	Dnepr River, Ukraine	Collected 1963	51.71	30.59						KX176794
SP317	United States	Alaska		64.20	-149.49						
SP738	Russia	Lugovskoya, Russia (Lugovskaya ul.?)		51.72	38.20						
SP739	Russia	Lugovskoya, Russia		51.72	38.20						
SP741	Russia	Shestakova, Russia		58.47	68.47						KX176765
SP743	Russia	Kochegur (near Shestakova, Russia)		58.47	68.47						
SP744	Russia	Kochegur (near Shestakova, Russia)		58.47	68.47						KX176766

Table S3: Published samples from Enk *et al* 2016 (reference 6)

ID/ Genbank ID	Region	State	Location	Museum Number	Latitude	Longitude	Radiocarbon Number	14C date	Sigma	intCal13 Median	Radiocarbon Date Reference
EEP41 KX02752	North America	MA	New Bedford Sound	HP2133	41.62	-70.88					
EEP42 KX027529	North America	MA	New Bedford Sound	HP2134	41.62	-70.88					
EEP43 KX027541	North America	CA	Santa Rosa Island	SBMNH27	33.97	-120.1					
EID02 JF912200	North America	AK	Upper Ikpikpuk R., North Slope	IK-99-70	70.47	-154.25	Beta #264909, Beta Analytic Inc., Miami, FL, USA	41510	480	44964	Enk et al. 2011
EID03 KX027564	North America	YU	Sulphur Creek	YPC136.0005	63.73	-138.83					
EID05 KX027567	North America	YU	Finning	YPC3.0256	63.83	-138.25	UCIAMS39115	28960	310	33125	Debruyne et al 2008
EID07 KX027566	North America	YU	Finning	YPC3.0229	63.83	-138.25					
EID09 KX027522	North America	AK	Upper Ikpikpuk R., North Slope	IK-99-235	69.37	-154.67	CAMS 91803	40870	820	44440	Debruyne et al 2008
EID10 KX027568	North America	YU	Hunker Creek	YPC5.0046	63.98	-139.03	UCIAMS41487	22430	140	26742	Debruyne et al 2008
EID11 KX027521	North America	AK	Upper Ikpikpuk R., North Slope	IK-98-1087	69.37	-154.67	CAMS 91795	>54000			Debruyne et al 2008
EID13 KX027524	North America	AK	Upper Ikpikpuk R., North Slope	IK-99-524	69.37	-154.67	CAMS 91811	>51000			Debruyne et al 2008
EID15 KX027556	North America	WY	nd	UW20579	43.22	-107.43	UCIAMS131716	38260	790	42451	Enk et al. 2015
EID18 KX027561	North America	YU	Old Crow; CRH-94	YP180.41	68.06	-139.78					
EID20 KX027560	North America	YU	Old Crow; CRH-94	YP180.40	68.06	-139.78					
EID23SEP03 KX027535	North America	IN	Near Goshen	HP1138	41.55	-85.93	BETA #92870	23050	180	27364	Dan
EID25 KX027542	North America	ME	Near Scarborough	HP1137	43.58	-70.32	OS-5636	12200	55	14091	Hoyle et al 2004
EID26 KX027537	North America	WY	Near Rawlins	UW6368	41.5	-107.63	Average of 4	11560	60	13392	Haynes et al 2013
EID27 KX027536	North America	NY	East Randolph Fish Hatchery	HP1134	42.15	-78.93	NOSAMS OS-93354	10350	45	12210	Feranec & Kozlowski 2012
EID30VEP84 KX027504	North America	AB	Bindloss	NMC17845	50.95	-110.13	TO-8514	10930	100	12825	Hills & Harington 2003

L200s KX027490	North America	SI	Taimyr Peninsula	2005/915	73.75	102	Beta-210777	27740	220	31501	Debruyne et al 2008
SED35 KX027493	North America	CO	Badger Creek	DMNS08	40.29	-106.45					
SEP01 KX027489	North America	SI	Taimyr Peninsula	2002/472	74.42	107.75	UCIAMS38677	>48800			Debruyne et al 2008
SEP02 KX027527	North America	MI	Clayton Township	HP1135	42.97	-83.97	UCIAMS131696	12450	40	14585	Enk et al. 2015
SEP06 KX027507	North America	IL	Gravel Pit near Clear Lake	ISM04	39.82	-89.53	NZA 32590	20550	100	24745	Enk et al. 2015
SEP07 KX027559	North America	IL	Wyanet	ISM07	41.37	-89.65	NZA 28851	15947	60	19237	Saunders et al. 2010
SEP11 KX027547	North America	NE	Red Willow Fauna, Rw-102	UNSM01	40.22	-100.37	UCIAMS131700	17070	70	20590	Enk et al. 2015
SEP17 KX027552	North America	NE	Richardson Co, Big Nemaha R.	UNSM15	40.12	-95.87					
SEP19 KX027503	North America	NE	South Fork Big Nemaha R.	UNSM21	40.07	-95.82	UCIAMS131707	13850	45	16770	Enk et al. 2015
SEP30 KX027511	North America	NE	Crappie Hole	UNSM34	41.2	-101.75	UCIAMS131713	23670	190	27778	Enk et al. 2015
SEP31 KX027512	North America	NE	Crappie Hole	UNSM35	41.2	-101.75	UCIAMS131714	23800	140	27863	Enk et al. 2015
SEP44 KX027494	North America	OR	Beak (Bear?) Creek	UCMP17	42.22	-122.72	UCIAMS131699	18510	100	22387	Enk et al. 2015
SEP50 KX027538	North America	MI	Morrison Lake Country Club, Saranac	HP1726	42.87	-85.2	BETA #282797	12320	50	14281	Dan
SEP52 KX027505	North America	SD	Near Brookings	ISM15	44.46	-96.88	CURL8895	12490	35	14716	Mandel 2004
SEP53 KX027500	North America	KY	Big Bone Lick	UNSM30	38.88	-84.75	UCIAMS131710	13215	40	15884	Enk et al. 2015
SEP58 KX027513	North America	CO	Dent	DMNS38	40.3	-104.8	Average of 3	10990	25	12827	Waters & Stafford 2007
SEP59 KX027514	North America	CO	Dent	DMNS40	40.3	-104.8	Average of 3	10990	25	12827	Waters & Stafford 2007
SEP61 KX027564	North America	YU	Ch'ijee's Bluff	YPC173.001	67.48	-139.92	UCIAMS41492	>45400			Debruyne et al 2008
SEP62 KX027495	North America	SI	Berelekh	Ber28	70.4	143.95	UCIAMS38670	12125	30	14011	Debruyne et al 2008
SEP63 KX027532	North America	YU	Dawson Area	NMC-49929	64.05	-139.42	AA17553	38600	2900	43496	Debruyne et al 2008
SEP64 KX027563	North America	YU	Quartz Creek	YPC130.0002	63.82	-139.03	UCIAMS39891	36690	810	41230	Debruyne et al 2008
SEP65 KX027523	North America	AK	Upper Ikpikpuk R., North Slope	IK-99-5001	69.37	-154.67	CAMS 91968	33530	340	37856	Debruyne et al 2008

SEP66 KX027531	North America	YU	Dawson Area	NMC-42292	64.05	-139.42	AA17535	37920	2700	42815	Debruyne et al 2008
SEP68 KX027492	North America	AK	Ester Creek	AM8744	64.83	-148	AA14896	16789	108	20256	Debruyne et al 2008
SEP69 KX027491	North America	AK	Cleary Creek	AM104	65.17	-147.5	AA14906	42764	1737	46455	Debruyne et al 2008
SID04-11 KX027533	North America	SI	Yakutia	2006/001-2	63.5	142.75	GrA-30727	41300	(+900-650)	44806	Debruyne et al 2008
SID36-10 KX027526	North America	RU	Yuribei R., Yamal Peninsula	HP1095	68.9	69.5	GrA-41246	41910	(+550-450)	45294	Fisher et al 2012
SSED27 KX027501	North America	KY	Big Bone Lick	UNSM31	38.88	-84.75	UCIAMS131711	13950	45	16926	Enk et al. 2015
SSED28 KX027502	North America	KY	Big Bone Lick	UNSM32	38.88	-84.75	UCIAMS131712	13860	40	16786	Enk et al. 2015
SSEP12 KX027548	North America	NE	Red Willow Fauna, Rw-102	UNSM02	40.22	-100.37	UCIAMS131701	12130	35	14014	Enk et al. 2015
SSEP18 KX027509	North America	NE	Crappie Hole	UNSM16	41.2	-101.75	UCIAMS131706	23590	130	27713	Enk et al. 2015
SSEP25 KX027498	North America	KY	Big Bone Lick	UNSM27	38.88	-84.75	UCIAMS131708	13985	45	16982	Enk et al. 2015
SSEP26 KX027499	North America	KY	Big Bone Lick	UNSM29	38.88	-84.75	UCIAMS131709	12930	40	15445	Enk et al. 2015
SSEP38 KX027517	North America	CO	Dent	DMNS47	40.3	-104.8	Average of 3	10990	25	12827	Waters & Stafford 2007
SSEP39 KX027519	North America	CO	nd	DMNS23	39.07	-105.13	UCIAMS131692	12475	40	14661	Enk et al. 2015
SSEP45 KX027540	North America	CA	San Antonio Creek	UCMP19	38.16	-122.53					
SSEP75 KX027508	North America	YU	Old Crow	CMNH40031	68.06	-139.78					
SVED74 KX027539	North America	CA	San Antonio Creek	UCMP04	38.16	-122.53	UCIAMS131697	19620	120	23642	Enk et al. 2015
SVEP05 KX027534	North America	IL	Near Pekin	ISM01	40.52	-89.72	UCIAMS131694	17510	70	21151	Enk et al. 2015
SVEP08 KX027545	North America	IL	Near Toledo	ISM09	39.28	-88.23					
SVEP09 KX027530	North America	IL	North LaSalle County	ISM12	41.55	-88.87	UCIAMS131695	12495	45	14724	Enk et al. 2015
SVEP13 KX027549	North America	NE	Red Willow Fauna, Rw-102	UNSM07	40.22	-100.37					
SVEP1478 KX027550	North America	NE	Red Willow Fauna, Rw-102	UNSM08	40.22	-100.37	UCIAMS131702	16160	80	19502	Enk et al. 2015
SVEP15 KX027551	North America	NE	Little Sand Pit near McCook, Rw-110	UNSM09	40.2	-100.5	UCIAMS131703	11585	35	13420	Enk et al. 2015

SVEP1679 KX027546	North America	NE	Trenton Reservoir	UNSM14	40.17	-101.07	UCIAMS131705	33670	450	37997	Enk et al. 2015
SVEP20 KX027553	North America	NE	Palisade Sand Pit	UNSM22	40.35	-101.42					
SVEP29 KX027510	North America	NE	Crappie Hole	UNSM33	41.2	-101.75					
SVEP32 KX027497	North America	NE	Biehl Farm	UNSM42	40.8	-99.65	UCIAMS131715	15200	60	18470	Enk et al. 2015
SVEP36 KX027543	North America	CO	Snowmass Site	SM3	39.21	-106.93					
SVEP37 KX027557	North America	WA	Wenas Creek	WAST_01	46.7	-120.55	WK-18064	13398	58	16122	Lubinski et al 2014
SVEP41 KX027515	North America	CO	Dent	DMNS43b	40.3	-104.8	Average of 3	10990	25	12827	Waters & Stafford 2007
SVEP43 KX027516	North America	CO	Dent	DMNS44	40.3	-104.8	Average of 3	10990	25	12827	Waters & Stafford 2007
SVEP48 KX027544	North America	OH	Near Cleves	ISM10	39.15	-84.75					
SVEP49 KX027520	North America	MI	North of Assyria	UM22798	42.52	-85.12	UCIAMS131693	11100	35	12985	Enk et al. 2015
SVEP5681 KX027525	North America	NE	La Sena	DMNS28b	40.38	-100.23	AA-6972	18440	145	22298	Holen 2006
SVEP57 KX027518	North America	CO	Dent	DMNS49	40.3	-104.8	Average of 3	10990	25	12827	Waters & Stafford 2007
SVEP72 KX027496	North America	NE	Beverly Gravel Pits	UNSM13	40.3	-101.03	UCIAMS131704	10650	30	12633	Enk et al. 2015
SVEP7380 KX027558	North America	WA	Whidbey Island	UCMP09	48.12	-122.58	UCIAMS131698	19200	120	23139	Enk et al. 2015
VEP82 KX027506	North America	NY	Near Chittenango	HP1133	43.05	-75.87	NOSAMS OS-93430	11250	65	13116	Feranec & Kozlowski 2012
VEP83 KX027562	North America	YU	Old Crow; CRH-11A	YP221.2	67.84	-139.85					

Table S4: Other previously published mitochondrial genomes used in this analysis

ID	Region	Location	Museum Number	Latitude	Longitude	Radiocarbon Number	14C date	Sigma	intCal13 Median	GenBank Accession	Reference
M13	Russia	Adams mammoth; Lena Delta, N-E Siberia		72.50	127.50		35800	1200	40418	EU153445	Gilbert et al. 2007
M15	Russia	Ayon Island, Russia		69.80	169.00	OxA 19605	13995	55	16996	EU153446	Gilbert et al. 2008
M18	Russia	Gydan Peninsula, Taimyr, West Siberia	KOS-W-1	72.09	79.35	OxA 17116	17125	70	20655	EU153447	Gilbert et al. 2007
M19	Russia	Yukagir Village, Yakutsk, Russia		71.87	140.58	GrN 28258, GrN 28259, GrN 24288	18560	50	22434	EU153448	Gilbert et al. 2008
M1	Russia	unknown; possibly Northern Yakutia (about 66 to 76N,106-160E)								EU153444	Gilbert et al. 2007
M20	Russia	Bolshoy Lyakhovsk(i)y Isl.	LDR-P72	73.64	142.89	OxA 19608	63500	infinite		EU153450	Gilbert et al. 2008
M21	Russia	Bolshoy Lyakhovsk(i)y Isl.	LDR-P73	73.21	143.60	OxA 19609	58000	infinite		EU153451	Gilbert et al. 2008
M22	Russia	Novosibirsk Islands N-E Siberia	LDR-P74	73.64	142.67	OxA 17111	50200	900		EU153452	Gilbert et al. 2007
M25	Russia	Bolshaya Chukochya River, Russia	F-0299	69.79	157.70	OxA 19610	59300	2700		EU153453	Gilbert et al. 2008
M26	Russia	Indigirka River, N-E Siberia	F-0308	68.60	147.06	OxA 17114	24740	110	28769	EU153454	Gilbert et al. 2007
M2	Russia	Jarkov mammoth, Taimyr Peninsula		73.32	105.40		20380	140	24507	EU153449	Gilbert et al. 2007
M3	Russia	Fishhook mammoth, Taimyr Peninsula		74.15	99.59		20620	70	24833	EU153455	Gilbert et al. 2007
M4	Russia	unknown; possibly Northern Yakutia (about 66 to 76N,106-160E)				OxA 17098	18545	70	22422	EU153456	Gilbert et al. 2007
M5	Russia	unknown; possibly Northern Yakutia (about 66 to 76N,106-160E)								EU153457	Giltert et al. 2007
M8	Russia	Dima mammoth, Magadan Region		62.67	147.93	OxA 17102	46900	700	46962	EU153458	Gilbert et al. 2007
NC007596	Russia	Berelyokh		71.00	145.00	KIA 25289	12170	50	14056	NC007596	Krause et al. 2006
Rogaev	Russia	Enmynveem		68.17	165.93		32750	1000	37068	DQ316067	Rogaev et al. 2006

Table S5: Details of individuals used in the *relaxed* and *strict* analyses.

ID	Unique reads mapped	Average Coverage	Cov3 Supp66_0.33 (relaxed)	Cov10 Supp90_0.2 (strict)	Clade
10643	36657	139.06	Y	Y	DE
10717	66970	289.31	Y	Y	2/A
10719	71765	315.98	Y	Y	DE
AlexKrim	19412	68.21	Y	Y	B2
SP1021	10132	36.32	Y	Y	DE
SP1022	78176	345.33	Y	Y	DE
SP1144	211184	818.96	Y	Y	B2
SP1145	100143	376.79	Y	Y	B2
SP1349	8526	31.76	Y	Y	2/A
SP1414	5396	16.14	Y	N	B2
SP1419	106672	412.9	Y	Y	DE
SP1420	1218	4.02	Y	N	2/A
SP1421	88251	409.63	Y	Y	DE
SP1422	23703	79.51	Y	Y	DE
SP1584	1024	3.31	N	N	-
SP167	5406	15.94	Y	N	B2
SP1781	540	1.95	N	N	-
SP1785	13392	39.66	Y	Y	B2
SP2013	11218	34.79	Y	Y	B2
SP2014	28926	95.29	Y	Y	B2
SP2015	8265	23.71	Y	N	B2
SP2016	10195	30.51	Y	Y	B2
SP2017	24778	88	Y	Y	B2
SP2218	37566	140.21	Y	Y	DE
SP2220	135075	633.16	Y	Y	DE
SP2222	22461	67.92	Y	Y	DE
SP2223	4943	16.59	Y	N	DE
SP2225	9664	40.31	Y	Y	DE
SP2283	7441	21.91	Y	N	B2
SP2284	10705	31.42	Y	N	B2
SP2293	5794	17.41	Y	N	B2
SP2299	1481	4.32	N	N	-
SP2300	751	2.17	N	N	-
SP2303	5496	16.3	Y	N	B2
SP2304	4377	12.79	Y	N	B2
SP2305	2370	6.83	Y	N	B2
SP2307	6553	19.29	Y	N	B2
SP2308	12577	37.29	Y	N	B2

SP2386	6553	31.05	Y	Y	DE
SP2387	21449	99.05	Y	Y	DE
SP2389	2021	9.66	Y	N	DE
SP2401	1575	7.11	Y	N	DE
SP2402	552	2.57	N	N	-
SP2411	41242	212.03	Y	Y	DE
SP2412	40623	201.38	Y	Y	B2
SP2415	116567	503.09	Y	Y	DE
SP2416	77000	416.61	Y	Y	B2
SP2453	2786	10.79	Y	N	B2
SP317	564	2.19	N	N	-
SP738	667	2.52	N	N	-
SP739	799	2.82	N	N	-
SP741	7697	23.34	Y	N	DE
SP743	731	2.1	N	N	-
SP744	6431	17.87	Y	N	DE
EEP41	2927	10.06	Y	N	C
EEP42	416	1.54	N	N	-
EEP43	601	1.63	N	N	-
EID02	73812	224.96	Y	Y	C
EID03	4987	16.54	Y	N	C
EID05	10262	32.61	Y	Y	C
EID07	1682	7.51	Y	N	C
EID09	806	3.09	N	N	-
EID10	3598	18.16	Y	Y	C
EID11	3810	12.9	Y	N	C
EID13	1601	6.91	Y	N	C
EID15	83069	220.15	Y	Y	C
EID18	1834	5.24	Y	N	B1
EID20	1392	4.58	Y	N	B1
EID23SEP03	20790	63.09	Y	Y	C
EID25	13543	46.57	Y	Y	C
EID26	8489	25.29	Y	Y	C
EID27	30981	90.94	Y	Y	C
EID30VEP84	5204	13.43	Y	N	C
L200s	11850	49.01	Y	Y	DE
SED35	367	1.18	N	N	-
1-Sep	148315	803.33	Y	Y	DE
2-Sep	10715	37.02	Y	Y	C
6-Sep	3163	10.85	Y	N	C
7-Sep	10127	32.08	Y	Y	C
11-Sep	14337	43.04	Y	Y	C
17-Sep	3218	8.5	Y	N	C

19-Sep	9461	25.67	Y	Y	C
30-Sep	3748	13.25	Y	N	C
Sep-31	6402	19.68	Y	Y	C
Sep-44	6961	24.3	Y	Y	C
Sep-50	11459	29.54	Y	Y	C
Sep-52	38058	149.89	Y	Y	C
Sep-53	38446	110.08	Y	Y	C
Sep-58	4403	15.65	Y	Y	C
Sep-59	7663	25.13	Y	Y	C
Sep-61	84565	300.07	Y	Y	C
Sep-62	48163	192.6	Y	Y	DE
Sep-63	66174	255.81	Y	Y	C
Sep-64	8869	34.23	Y	Y	DE
Sep-65	13220	52.92	Y	Y	DE
Sep-66	68805	241.73	Y	Y	B1
Sep-68	47614	179.31	Y	Y	DE
Sep-69	58248	239.46	Y	Y	C
SID04-11	10868	44.62	Y	Y	A
SID36-10	46331	163.01	Y	Y	B2
SSED27	4329	10.05	Y	N	C
SSED28	5391	12.95	Y	N	C
SSEP12	14133	41.18	Y	Y	C
SSEP18	11513	40.72	Y	Y	C
SSEP25	5042	12.17	Y	N	C
SSEP26	2922	7.12	Y	N	C
SSEP38	20118	54.94	Y	Y	C
SSEP39	32664	98.51	Y	Y	C
SSEP45	13303	32.12	Y	Y	C
SSEP75	6236	23.12	Y	N	B1
SVED74	5022	13.29	Y	N	C
SVEP05	7469	18.62	Y	N	C
SVEP08	3040	7.29	Y	N	C
SVEP09	12779	35.53	Y	Y	C
SVEP13	1168	2.95	N	N	-
SVEP1478	5091	13.4	Y	N	C
SVEP15	7065	20.06	Y	N	C
SVEP1679	3261	8.3	Y	N	C
SVEP20	9061	24.36	Y	Y	C
SVEP29	2455	7.65	Y	N	C
SVEP32	4749	13.58	Y	N	C
SVEP36	2027	4.49	N	N	-
SVEP37	5100	13.45	Y	N	C
SVEP41	15995	50.88	Y	Y	C

SVEP43	20268	62.94	Y	Y	C
SVEP48	7276	18.4	Y	N	C
SVEP49	9328	23.68	Y	Y	C
SVEP5681	3927	12.81	Y	N	C
SVEP57	12996	37.92	Y	Y	C
SVEP72	3511	8.45	Y	N	C
SVEP7380	1668	4.16	Y	N	C
VEP82	129063	405.48	Y	Y	C
VEP83	1015	3.09	N	N	-

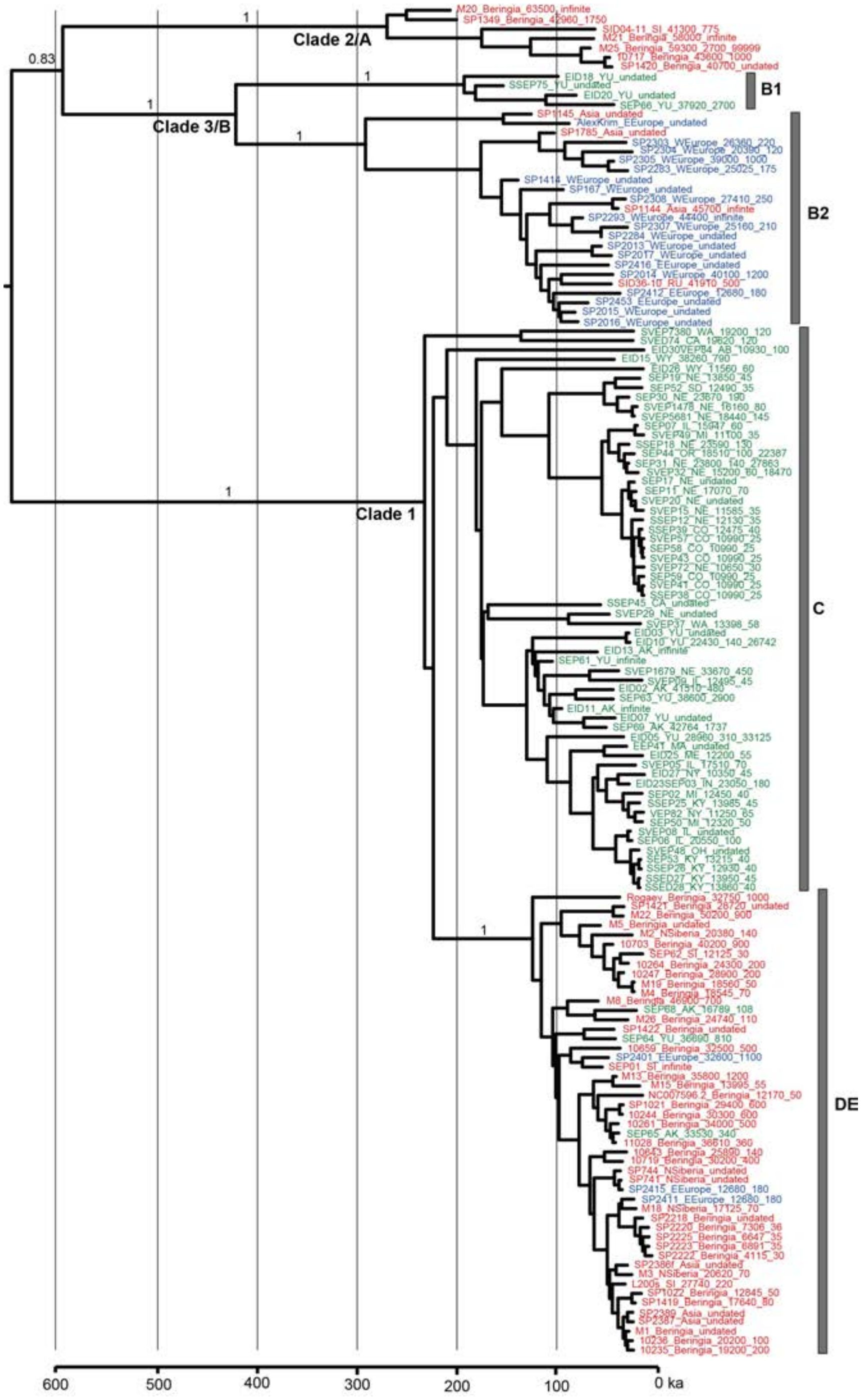

Figure S1. Maximum Clade Credibility (MCC) tree of the *relaxed* data set with the tip-dating method. Posterior probabilities are provided at each internal branch. The scaled axis of time is offset by 4,641, the calibrated age of the youngest sample. Samples labeled in red are from Asia, those in green are from North America and those in blue are sampled from Europe.

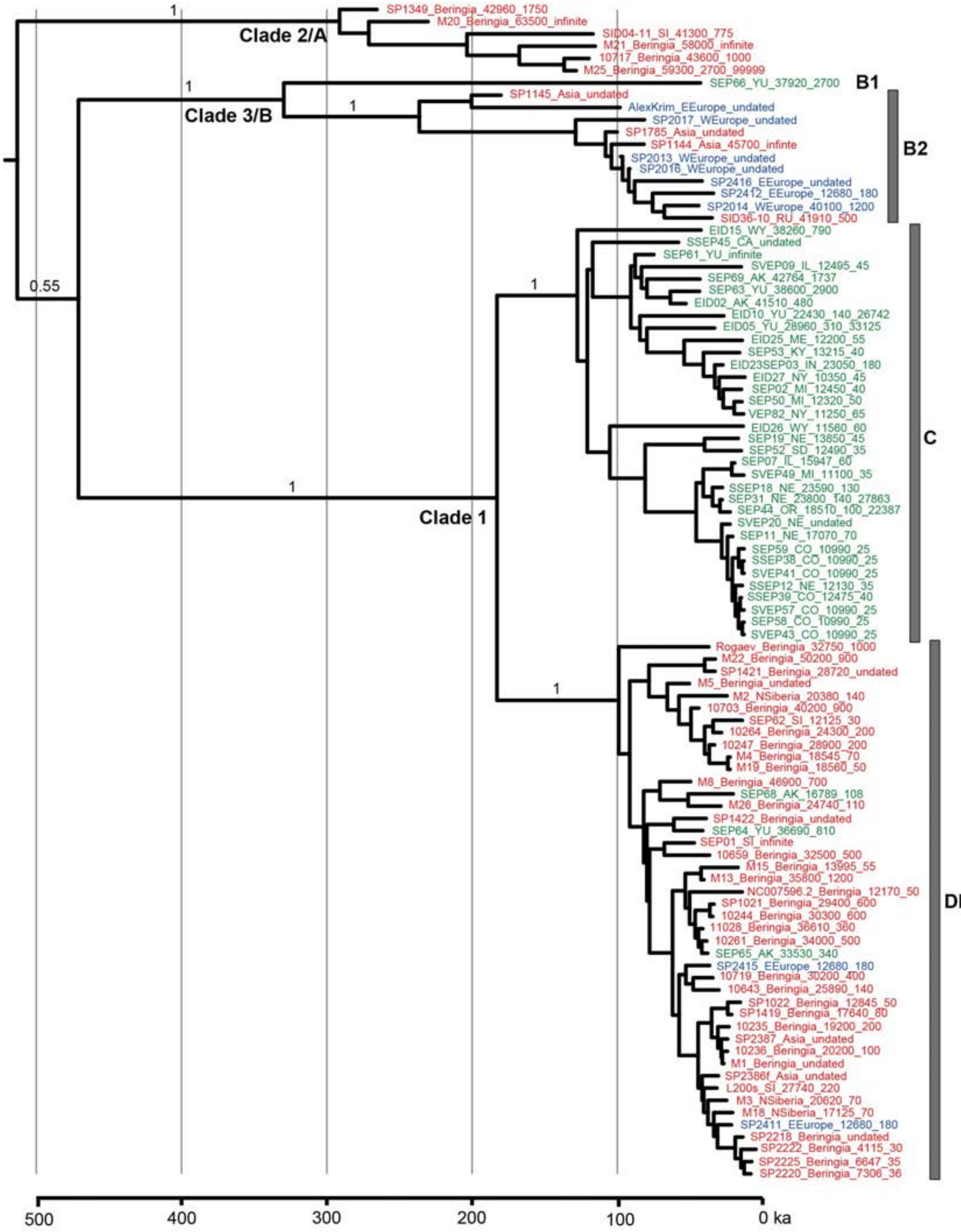

Figure S2. MCC tree of the *strict* data set with the tip-dating method. Descriptions are the same as in Fig. 1.

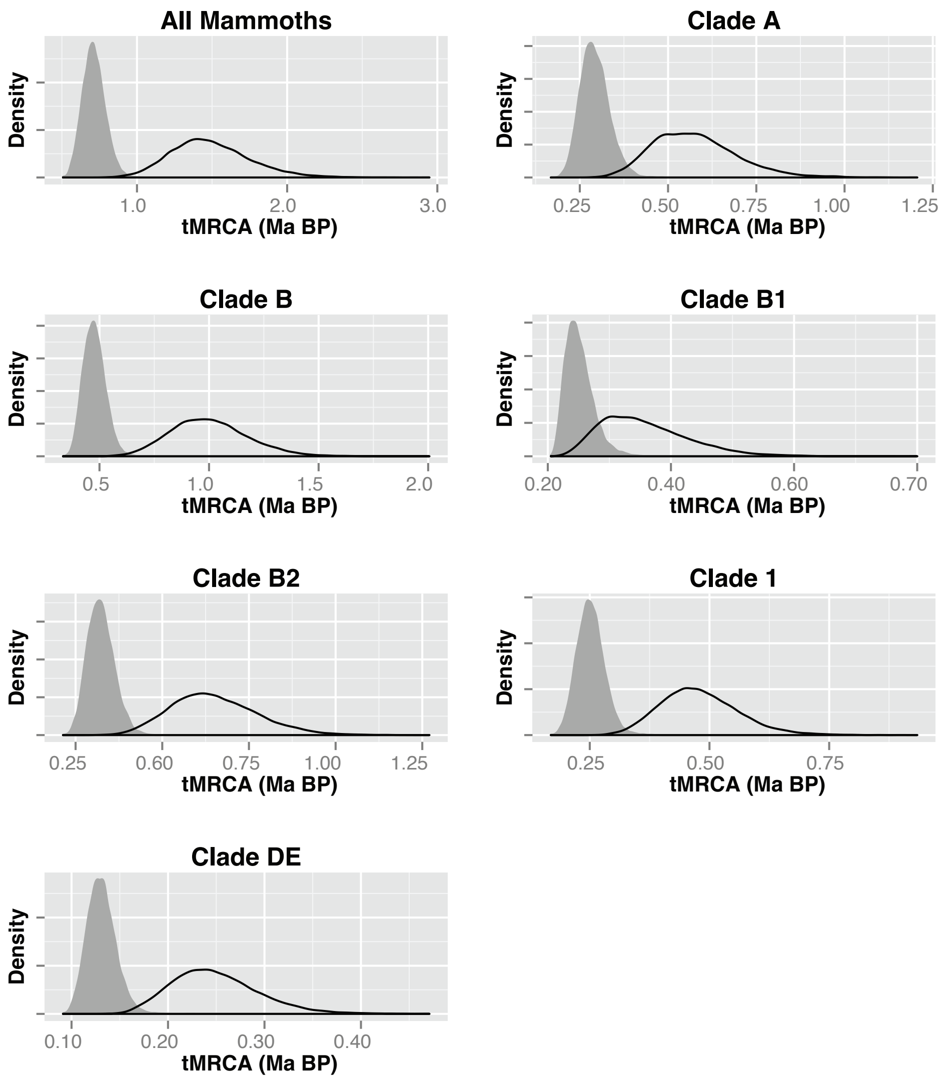

Figure S3. Gaussian densities (95% highest posterior densities) of estimated tMRCAs for each major clade using the two dating methods. Results of the tip-dating method are given as shaded gray distributions, and results from the root-and-tip-dating method are shown as a black line.
